# Diagnostic accuracy of TB-LAMP and smear microscopy for pulmonary TB in active case finding

**DOI:** 10.5588/ijtldopen.25.0557

**Published:** 2026-03-13

**Authors:** A. Shimouchi, R.S. Gopali, B. Maharjan, Y. Shrestha, Y. Hirano, N.P. Shah, P. Ghimire, P. Shrestha, K. Okada

**Affiliations:** 1 Japan Anti-Tuberculosis Association (JATA), Nepal Office, Kathmandu, Nepal;; 2 Research Institute of Tuberculosis, JATA, Tokyo, Japan;; 3 Japan-Nepal Health and Tuberculosis Research Association, Kathmandu, Nepal;; 4 Department of International Program, JATA, Tokyo, Japan;; 5 National Tuberculosis Control Center, Thimi, Nepal;; 6 National Academy of Medical Sciences, Bir Hospital, Kathmandu, Nepal.

**Keywords:** tuberculosis, Nepal, presumptive TB, early case detection, bacteriological test, symptomatic screening, X-ray screening

## Abstract

**BACKGROUND:**

In Nepal, most sputum tests are done with smear microscopy. However, in order to increase case detection by active case finding (ACF), more sensitive bacteriological test should be used.

**METHODS:**

Presumptive TB cases aged five and over detected by symptomatic screening and X-ray screening for pulmonary TB were included in the study. Smear microscopy, loop-mediated isothermal-amplification for TB (TB-LAMP), and Xpert MTB/RIF (Xpert) were performed as part of routine TB diagnostic procedure in ACF. We compared the diagnostic accuracy of TB-LAMP and smear microscopy by using Xpert as the microbiological reference standard.

**RESULTS:**

Among 653 samples, sensitivity, specificity, and kappa value of smear microscopy and TB-LAMP were 30.5% (95% confidence interval [CI]: 21.6–41.1), 99.8% (95% CI: 99.0–99.9), 0.43 (95% CI: 0.36–0.49) and 72.0% (95% CI: 61.4–80.5), 99.7% (95% CI: 98.7–99.9), 0.80 (95% CI: 0.73–0.88), respectively. Sensitivity of TB-LAMP (72.0%) was 41.5% higher than that of smear microscopy (30.5%) (*P* < 0.01). Sensitivity of TB-LAMP and smear microscopy was high for multibacillary TB. Sensitivity of TB-LAMP was substantially higher than that of smear microscopy for paucibacillary TB.

**CONCLUSION:**

Sensitivity of TB-LAMP was much higher than that of smear microscopy in ACF. TB-LAMP is recommended to replace smear microscopy in ACF to increase the detection of bacteriologically confirmed pulmonary TB.

TB is a major public health concern in Nepal. In 2023, the WHO estimated 68,000 new TB cases with the rate of 229 (126–355) per 100,000 population. However, only 36,881, half of the estimated incident cases, were notified.^1^ However, smear microscopy with Ziehl–Neelsen staining is the most commonly used initial diagnostic tool. Overall smear positivity has been reduced from 7.6% (13,170/172,588) in 2016 to 3.3% (12,761/329,234) in 2023.^2^ To improve case detection rate, Xpert MTB/RIF (Xpert; Cepheid, Sunnyvale, USA), endorsed by the WHO,^3^ was first introduced in 2011 in specific populations recommended by the WHO: such as children <15 years and people living with HIV.^4^ Later on, the target had been expanded to the general population and Xpert was available in 117 health facilities in 2024.^2^ However, the problems still remain, such as high device cost, the need of stable electricity supply, and continuous instrument maintenance for further expansion.

The loop-mediated isothermal-amplification for TB (TB-LAMP) is a commercially available, manual molecular TB detection method manufactured by Eiken Chemical Company, Tokyo, Japan.^5^ TB-LAMP assay does not require sophisticated instruments but requires only similar biosafety level with that of smear microscopy. With trained laboratory technicians, it takes 60–90 min to complete all procedures and it generates a fluorescent product that can be seen under UV lights with the naked eye. As another advantage, it can test up to 14 samples per batch.^5,6^ And more importantly, pooled sensitivity of TB-LAMP was 78% and 15% higher than that of smear microscopy of 63% in diagnosis of symptomatic pulmonary TB in reference to mycobacterial culture according to the WHO policy guidance based on the reports from intermediate- to high-TB-burden countries.^6,7^ Furthermore, new WHO guidelines recommend that TB-LAMP can be used for adolescents (aged 10–19) as well as adults.^3^ In Nepal, symptomatic screening was introduced as active case finding (ACF) in 2016 and is currently carried out in 42 out of 77 districts in the country. In addition, ACF with X-ray was introduced in 2022 and has been expanded as nation-wide programme in 2024–2025.

This study aims to evaluate the diagnostic accuracy of TB-LAMP for pulmonary TB in ACF against smear microscopy using Xpert MTB/RIF as the microbiological reference standard.

## METHODS

The study was conducted at Japan-Nepal Health and Tuberculosis Research Association (JANTRA) laboratory in Kathmandu City from November 2023 to January 2025. The laboratory received sputum samples from chest X-ray screening in community and congregate settings conducted by JANTRA and symptomatic screening in various health facilities mentioned below.

### Study participants

Participants were persons aged five and over and were considered as presumptive TB who were categorised in different settings as follows:

### Presumptive TB of X-ray screening

Target age group of X-ray screening was persons aged 15 and over in general community and high-risk population including residents in slum areas, and congregate settings such as boarding schools, factories, and monasteries. In addition, household contacts aged five and over of bacteriologically confirmed TB patients were eligible. Persons with chest X-ray findings suggestive of TB regardless of symptoms were considered as presumptive TB. Their sputum samples were taken and brought to JANTRA laboratory.

### Presumptive TB of symptomatic screening

A person aged five and over with any one or more of the following symptoms was considered as presumptive TB according to the government guideline^8^: cough for 2 weeks or more, coughing sputum with or without blood, fever, night sweats, loss of appetite, and weight loss. Sputum samples of presumptive TB cases were collected from health facilities and carried by outreach workers or other staff to JANTRA laboratory.

Procedures to identify presumptive TB among different target groups were as follows:

1) Contact tracing: When bacteriologically confirmed TB patients were registered at TB treatment centres, health workers of urban health clinics and Female Community Health Volunteers instructed household members to obtain sputum samples from them if they had TB symptoms.
2) Public–Private Mix^9–11^: At the pharmacies engaged through hub and spoke model for TB case finding, people were identified as presumptive TB by symptomatic screening. They were requested to produce the sputum sample on the spot or referred for clinical examination at private clinics.
3) Pay for performance: When presumptive TB was identified at private clinics or private hospitals, sputum samples were taken for examination.^9^
4) FAST Strategy^12^: FAST is an intervention implemented in public and private hospitals. FAST mobilisers conducted cough screening at selected outpatient departments (OPDs) and service areas. If they found the patients with cough, they performed screening for other symptoms suggestive of TB, and then referred them to doctors for confirmation of presumptive TB.

### Sputum sample collection and transportation

For both X-ray screening and symptomatic screening, one sputum sample per presumptive TB was collected and transported to JANTRA laboratory for diagnosis.

### Sputum smear microscopy

Sputum sample was smeared directly on a slide and subjected to Ziehl–Neelsen staining for acid-fast bacilli and examined.^13^

### TB-LAMP assay

The TB-LAMP assay was performed according to the manufacturer’s instructions. In brief, 60 μL of sputum sample was pipetted into heating tubes and incubated at 90°C for 5 min for lysis. The purified DNA was eluted from the absorbent tube and transferred into injection caps. After mixing with lyophilised reagents, the amplification mixture was incubated at 67°C for 40 min. The results were detected using ultraviolet fluorescence. The turnaround time of TB-LAMP was 60 min, and up to 14 specimens could be handled per batch.

### Xpert MTB/RIF and Xpert MTB/RIF Ultra assay

The Xpert assay was performed according to the manufacturer’s instructions. In brief, collected sputum sample was mixed with the sample reagent at a 2:1 ratio, incubated at room temperature for 15 min. A 2 mL aliquot of the processed sample was then transferred into the Xpert MTB/RIF cartridge using a sterile plastic pipette and loaded onto the Xpert instrument. The analysis software automatically classified the results. The turnaround time for the Xpert MTB/RIF was approximately 125 min, enabling the detection of *Mycobacterium tuberculosis* (MTB) and rifampicin resistance. The Xpert instrument used in this study was capable of simultaneously processing up to four tests.

### Samples eligible for the study

Xpert MTB/RIF cartridges were used from November to December 2024 and Xpert MTB/RIF Ultra cartridges from January 2024 to January 2025. However, samples collected from January to May 2024 were not eligible for the study because Xpert was not tested for all cases due to shortage of Xpert cartridges. However, from November to December 2023 and from June 2024 to January 2025, all consecutive samples were tested by three methods without exception and included for the study.

### Data analysis

We compared the diagnostic accuracy of TB-LAMP and smear microscopy by measuring sensitivity, specificity, positive predictive value (PPV), negative predictive value (NPV), and kappa value and their exact binomial 95% confidence intervals (CIs) in bacteriological reference of Xpert. Concordance between two tests was assessed using the κ coefficient. κ values of 0.41–0.60 indicate moderate agreement, and 0.61–0.80 as substantial agreement. *P* value of less than 0.05 was considered statistically significant.

### Ethical statement

The protocol for this study including the aspect of ethical conduct was reviewed and approved by Nepal Health Research Council (NHRC), Government of Nepal, Kathmandu, Nepal (Reference Number: 305), and the Ethical Committee of the Research Institute of Tuberculosis, Japan Anti-Tuberculosis Association (RIT/JATA), Tokyo, Japan (Approval Number: RIT/TRB 2025-2).

## RESULTS

A total of 653 sputum samples of presumptive TB were successfully tested with smear microscopy, TB-LAMP, and Xpert assay (MTB/RIF 91, Ultra 562). However, one sample was excluded from the analysis because the result was smear microscopy: negative, TB-LAMP: negative, and Xpert: error. More than half (55%) of them were male. Age group of less than 15, 15–64, and 65 and over consisted of 5.5%, 69.8%, and 24.7%, respectively. The positivity of smear microscopy, TB-LAMP, and Xpert was 4.0%, 9.3%, and 12.6%, respectively (Table 1).

**Table 1.** Demographic characteristics and test results of presumptive TB cases according to mode of active case finding.

Characteristics	Symptomatic screening	X-ray screening	Total
	n = 420	n = 233	n = 653
	n (%)	n (%)	n (%)
Sex			
Female	197 (46.9)	97 (41.6)	294 (45.0)
Male	223 (53.1)	136 (58.4)	359 (55.0)
Age			
5–14	12 (2.9)	24 (10.3)	36 (5.5)
15–64	308 (73.3)	148 (63.5)	456 (69.8)
65 and over	100 (23.8)	61 (26.2)	161 (24.7)
Smear microscopy result			
Positive	14 (3.3)	12 (5.2)	26 (4.0)
Negative	406 (96.7)	221 (94.8)	627 (96.0)
TB-LAMP result			
Positive	32 (7.6)	29 (6.8)	61 (9.3)
Negative	388 (92.4)	204 (93.2)	592 (90.7)
Xpert MTB/RIF and Ultra result			
Positive	41 (10.0)	41 (18.3)	82 (12.6)
Negative	379 (90.0)	192 (81.7)	571 (87.4)

In addition to the above data, there was one sample which showed microscopy negative, TB-LAMP negative, and Xpert error. It was excluded from the above analysis.

TB-LAMP = loop-mediated isothermal-amplification for TB.

Diagnostic performance of TB-LAMP in comparison with smear microscopy in reference to Xpert MTB/RIF and Ultra is given in Table 2. Sensitivity of smear microscopy and TB-LAMP was 30.5% (95% CI: 21.6–41.1) and 72.0% (95% CI: 61.4–80.5), respectively. The specificity, PPV, NPV, and kappa value of smear microscopy and TB-LAMP were 99.8% (95% CI: 99.0–99.9), 96.2% (95% CI: 81.1–99.3), 90.9% (95% CI: 88.4–92.9), and 0.43 (95% CI: 0.36–0.49) and 99.7% (95% CI: 98.7–99.9), 96.7% (95% CI: 88.8–99.1), 96.1% (95% CI: 94.2–97.4), and 0.80 (95% CI: 0.73–0.88), respectively. Sensitivity of TB-LAMP (72.0%) is 41.5% higher than that of smear microscopy (30.5%) (*P* < 0.01). And the Kappa value of TB-LAMP (0.80) and that of smear microscopy (0.43) are considered as substantial agreement and moderate agreement, respectively, and difference is statistically significant (χ^2^, *P* < 0.05) (Table 2).

**Table 2.** Diagnostic performance of TB-LAMP in comparison with smear microscopy in reference to Xpert MTB/RIF and Ultra.

Xpert MTB/RIF and Ultra		+	−	Total	Sensitivity (95% CI)	Specificity (95% CI)	PPV (95% CI)	NPV (95% CI)	Kappa value (95% CI)
Smear microscopy	+	25	1	26	30.5% (21.6–41.1)	99.8% (99.0–99.9)	96.2% (81.1–99.3)	90.9% (88.4–92.9)	0.43 (0.36–0.49)
	−	57	570	627					
TB-LAMP	+	59	2	61	72.0% (61.4–80.5)	99.7% (98.7–99.9)	96.7% (88.8–99.1)	96.1% (94.2–97.4)	0.80 (0.73–0.88)
	−	23	569	592					
	Total	82	571	653					

TB-LAMP = loop-mediated isothermal-amplification for TB; CI = confidence interval; PPV = positive predictive value; NPV = negative predictive value.

In subgroup analysis, sensitivities of TB-LAMP for smear-positive and smear-negative TB were 100% (25/25) and 59.6% (34/57), respectively (Table 3). The difference was statistically significant (χ^2^, *P* < 0.001). Sensitivity of TB-LAMP with reference to Xpert MTB/RIF and Xpert MTB/RIF Ultra were 80.0% (4/5) and 71.4% (55/77), respectively. Specificity of TB-LAMP with reference to these assays were 100% (53/53) and 99.6% (516/518), respectively. Both were statistically insignificant (Table 4).

**Table 3.** Comparison of positivity of smear microscopy and TB-LAMP among Xpert MTB/RIF and Ultra positive samples.

	TB-LAMP				
		+ n (%)	− n (%)	Total	χ^2^ *P* value
Smear microscopy	+	25 (100)	0 (0)	25	<0.001
	−	34 (59.6)	23 (40.4)	57	
	Total	59 (72.0)	23 (28.0)	82	

TB-LAMP = loop-mediated isothermal-amplification for TB.

**Table 4.** Sensitivity and specificity of TB-LAMP with reference to Xpert MTB/RIF and Xpert MTB/RIF Ultra.

		TB-LAMP		Sensitivity	χ^2^ *P* value
		+	−		
Xpert MTB/RIF	+	4	1	80.0%	0.679
Xpert MTB/RIF Ultra	+	55	22	71.4%	

		TB-LAMP		Specificity	χ^2^ *P* value
		+	−		
Xpert MTB/RIF	−	0	53	100%	0.650
Xpert MTB/RIF Ultra	−	2	516	99.6%	

TB-LAMP = loop-mediated isothermal-amplification for TB.

The Figure shows positivity of TB-LAMP and smear microscopy by bacillary load of Xpert MTB/RIF and Ultra. Positivity of TB-LAMP was 100% in high and medium load. However, it was 77% in low and 63% in very low load. Positivity of smear microscope was considerably high as 93% in high and 85% in medium load. However, it was very low of 4% in low and 0 in very low. Both TB-LAMP and smear microscopy were negative in trace.

**Figure.**
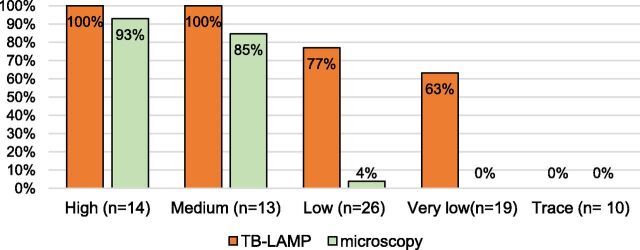
Positivity of TB-LAMP and smear microscopy by bacillary load of Xpert in active case finding (n = 82). TB-LAMP = loop-mediated isothermal-amplification for TB.

## DISCUSSION

This study showed that sensitivity and kappa value of TB-LAMP is significantly higher than those of smear microscopy, when Xpert MTB/RIF and Ultra was used as the microbiological reference standard. Sensitivities of TB-LAMP for smear-positive and smear-negative TB were 100% and 59.6%, respectively. They were in the same range of sensitivities of TB-LAMP for smear-positive TB (92.1%–99.1%) and smear negative TB (46.6%–72.7%) reported in the studies in intermediate- to high-TB-burden countries when participants were OPD patients with TB symptoms, that is, detected by passive case finding (PCF) and culture was used as reference.^14–18^ Then, we confirmed the finding of higher sensitivity of TB-LAMP than that of smear microscopy in ACF, which had already been demonstrated among symptomatic presumptive TB in PCF.

In our study, ACF with X-ray was initiated by health service-providers. We invited participants regardless of symptoms. However, ACF with symptomatic screening in health facilities including pharmacies involved patient’s decision to attend these facilities. In this connection, the study in Kenya^19^ showed that TB was diagnosed through symptomatic screening mainly by Xpert in 20.3% in health facilities and 3.4% in communities. The difference in positivity explained that symptomatic persons visiting health facilities had more severe symptoms than those symptomatic persons who stayed in community. On the other hand, in our study, smear positivity of presumptive TB detected by symptomatic screening in health facilities was 3.3%, lower than that of X-ray screening in community and congregate settings of 5.2%. It may indicate that symptomatic screening in health facilities detected TB disease at an early stage probably because majority of general population in Kathmandu City utilised health facilities at the earliest without monetary constraints. Comparatively, X-ray screening was conducted in community, especially for high-risk population, which leads to higher positivity of bacteriological tests.

The novelty of this study is that we showed that sensitivity of TB-LAMP (72.0%) in ACF was similar to the level of sensitivity of TB-LAMP (78%) in PCF, but that of smear microscopy (30.5%) in ACF was much lower than pooled sensitivity (65%) of studies in PCF in the past. Consequently, the difference of sensitivity of the two methods was 41.5% in our study, larger than the difference of 13% among pooled sensitivity of past studies. In this study, presumptive TB would include more mild forms of diseases, that is, asymptomatic cases in X-ray screening and cases with single TB symptom in symptomatic screening. The Figure clearly shows that sensitivity of TB-LAMP and smear microscopy was the same for multibacillary (high and medium load) TB, while sensitivity of TB-LAMP was much higher than that of smear microscopy for paucibacillary (low and very low load) TB. In the past studies, definition of presumptive TB was cough and one of other TB symptoms.^14–18^ Therefore, relatively high sensitivity of smear microscopy (65%) was due to higher proportion of multibacillary TB in the studies. It is logical that more TB symptoms indicate more advanced in disease condition reflected in the higher proportion of smear positivity. As the evidence, a systematic review of literatures on early detection of TB summarised the findings as follows: screening individuals for TB helps facilitate early detection of the disease.^20^ In other words, through ACF, more paucibacillary pulmonary TB cases are detected; hence, difference of sensitivities of TB-LAMP and smear microscopy is larger in ACF.

It is obvious that incremental budget is needed for human resource and cost for transportation of sputum samples for ACF with symptomatic screening and the operation of X-ray screening by technical team. Therefore, a cost-effective study should be conducted to compare TB-LAMP and smear microscopy as initial bacteriological test in ACF.

In addition to diagnostic accuracy, operational feasibility is an important consideration for introducing TB-LAMP into ACF. Compared with smear microscopy, TB-LAMP requires specified human resources and training, as laboratory staff must be familiar with nucleic acid amplification procedures and batch-based workflows. However, the infrastructure needs are modest: the assay requires only a heating block, a UV lamp, and stable electricity. Taken together, TB-LAMP offers a balance of sensitivity, feasibility, and affordability that makes it a practical replacement for smear microscopy in ACF programmes where Xpert expansion is constrained by higher cost and maintenance requirements.

A limitation of the study is the use of Xpert as a reference standard. To obtain a more accurate assessment, culture or quantitative polymerase chain reaction with TB-LAMP or Xpert should be used. However, Xpert is much easier and practical to use as reference than culture in the laboratory without special biosafety facilities. HIV test for TB patient is currently done at the TB registration at health facilities as national policy. But at the time of reception of sputum samples at the laboratory, HIV test had not been done yet. And the test result was not obtained retrospectively from health facilities. Thus, we could not compare test results with HIV status. However, according to the national statistics,^3^ 95.4% of TB patients were tested on HIV infection and positivity was 0.6% in 2023. Therefore, HIV positivity among TB patients in our study would not affect the result.

## CONCLUSION

The sensitivity of TB-LAMP was much higher than that of smear microscopy in ACF. Therefore, TB-LAMP is recommended to replace smear microscopy in ACF to increase the detection of bacteriologically confirmed pulmonary TB.
